# Analysis of the cross-talk of Epstein–Barr virus-infected B cells with T cells in the marmoset

**DOI:** 10.1038/cti.2017.1

**Published:** 2017-02-10

**Authors:** Jordon Dunham, Nikki van Driel, Bart JL Eggen, Chaitali Paul, Bert A ‘t Hart, Jon D Laman, Yolanda S Kap

**Affiliations:** 1 Department of Immunobiology, Biomedical Primate Research Centre, Rijswijk, The Netherlands; 2 Department of Neuroscience, University Groningen, University Medical Center, Groningen, The Netherlands

## Abstract

Despite the well-known association of Epstein–Barr virus (EBV), a lymphocryptovirus (LCV), with multiple sclerosis, a clear pathogenic role for disease progression has not been established. The translationally relevant experimental autoimmune encephalomyelitis (EAE) model in marmoset monkeys revealed that LCV-infected B cells have a central pathogenic role in the activation of T cells that drive EAE progression. We hypothesized that LCV-infected B cells induce T-cell functions relevant for EAE progression. In the current study, we examined the *ex vivo* cross-talk between lymph node mononuclear cells (MNCs) from EAE marmosets and (semi-) autologous EBV-infected B-lymphoblastoid cell lines (B-LCLs). Results presented here demonstrate that infection with EBV B95-8 has a strong impact on gene expression profile of marmoset B cells, particularly those involved with antigen processing and presentation or co-stimulation to T cells. At the cellular level, we observed that MNC co-culture with B-LCLs induced decrease of CCR7 expression on T cells from EAE responder marmosets, but not in EAE monkeys without clinically evident disease. B-LCL interaction with T cells also resulted in significant loss of CD27 expression and reduced expression of IL-23R and CCR6, which coincided with enhanced IL-17A production. These results highlight the profound impact that EBV-infected B-LCL cells can have on second and third co-stimulatory signals involved in (autoreactive) T-cell activation.

Epstein–Barr virus (EBV), a causative agent of classical infectious mononucleosis, is a γ1-herpes virus and the human representative among a larger group of primate lymphocryptoviruses (LCVs).^1, 2^ Despite numerous lines of evidence indicating an association between EBV and autoimmune conditions such as multiple sclerosis (MS), an exact pathogenic role in autoimmune diseases is unclear.^3^ As non-human primates are naturally infected with EBV-related LCV, they provide potentially relevant animal models in which the relationship between EBV and autoimmunity can be explored. The experimental autoimmune encephalomyelitis (EAE) model in common marmosets (*Callithrix jacchus*) is a translationally relevant model of the autoimmune neurological disease MS.^4^ The common marmoset is also host to a B-cell transforming lymphocrypto/γ1-herpes virus, CalHV-3 (callithrichine herpes virus 3), which is considered to be homologous to EBV.^5^ Via reverse translation analysis of therapies that did (anti-CD20 monoclonal antibodies (mAbs))^6^ or did not (atacicept)^7^ show a beneficial effect in MS clinical trials, we discovered a new pathogenic role of B cells in the activation of myelin oligodendrocyte glycoprotein (MOG)-specific T cells in this model.^8^ In brief, we discovered that the opposite clinical effect of these treatments might be explained by the differential depletion of B cells infected with CalHV-3.^9^ These and other findings led us to postulate that LCV-infected B cells have a core pathogenic role in the marmoset EAE model that is endowed by the virus infection.^10^

The aim of the current study was to gain deeper insight into the cross-talk between LCV-infected B cells and autoreactive T cells isolated from marmosets sensitized against MOG34–56 formulated with the mineral oil incomplete Freund's adjuvant (IFA). This formulation induces a progressive MS-like disease characterized by MS-like pathology in the white and cortical gray matter of brain and spinal cord.^11, 12^ We first analyzed using RNAseq which B-cell genes relevant for T–B communication were expressed at higher level after LCV infection. For these experiments, we used EBV from the cell line B95-8. In addition, we tested the role of several receptor–ligand pairs using mAbs.

The results demonstrate that interactions between LCV-infected B cells and lymph node-derived T cells from MOG34–56/IFA-immunized marmosets lead to profound phenotypic alterations of T cells. These data may be interpreted as an attempt of the EBV-infected B cells to escape the immune system by phenotypically altering the T cells in critical markers related to homing, Th17 responses, development and function that may partially explain the temporal association between EBV and autoimmune diseases.

## Results

### EBV infection of B cells alters stimulation signals to T cells

**Figure 1:**
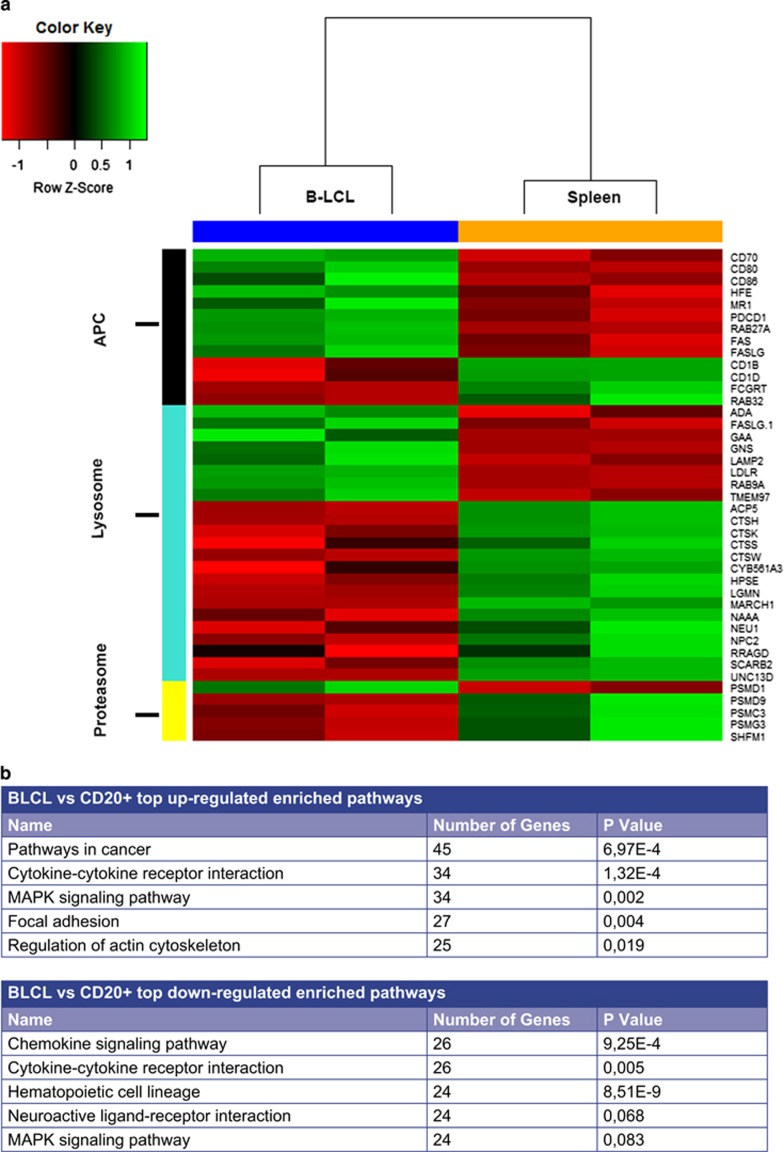
EBV infection alters critical markers involved in antigen presentation and co-stimulation of the B cell. RNA sequencing was performed on spleen-derived CD3^−^CD20^+^ B cells from immunologically healthy marmosets (*n*=2) and marmoset B-LCLs (*n*=2), with 10 million high-quality uniquely mapped reads were obtained per sample, to determine the effect of EBV infection on gene expression involved in antigen presentation and co-stimulation pathways. EBV infection resulted in differential expression (FDR<0.05, logFC=±1) in pathways associated with cell growth/survival functions, metabolic processes and antigen presentation/co-stimulation. (**a**) Shown is a heatmap depicting genes associated with the selected categories antigen presentation, lysosome and proteasome (processing) function chosen from the list of differentially expressed genes. Tabular data for these genes are in Supplementary Table 1. The scaled expression value (*Z* score) is displayed in red-green color scheme with red indicating lower expression and higher expression in green. (**b**) Depicted are the top five up- and downregulated pathways.

RNA sequencing was used to determine the impact of EBV infection on marmoset B cells (B-lymphoblastoid cell line, B-LCL) gene expression in comparison to spleen-derived CD3^−^CD20^+^ B cells. A principle component analysis was performed to determine the similarity between biological replicates and differences between samples conditions (data not shown). A differential gene expression analysis was performed to determine the genes that were differentially expressed between B-LCLs and CD20^+^ B cells and to identify pathways and overrepresented categories (Figure 1). In total, LCV infection resulted in increased expression of 2860 genes (false discovery rate (FDR)<0.05; logFC>+1) and reduced expression of 2608 genes (FDR<0.05; logFC<−1). Shown in the table of Figure 1b is a list of top upregulated and top downregulated enriched pathways. As could be expected, we also observed a clear enrichment of cell cycle-related genes, and altered the expression of apoptosis genes, which is consistent with the fact that B-LCLs are immortalized lines (data not shown).

We were particularly interested in the expression of genes related to T-cell activation to determine how EBV influences antigen-presenting cell (APC) functions of B cells. We observed differential expression of many genes related to antigen presentation and processing pathways, particularly those of co-stimulation and peptide processing (Figure 1). Shown in Figure 1 is a heatmap of differentially expressed genes related to antigen presentation/co-stimulation, with noted upregulation in critical surface markers such as CD70, CD80, CD86, PCDC1 (PD-1) and both FAS (CD95)/FASL (CD95L) being observed. Furthermore, expression of a number of genes involved in peptide processing via the vacuolar route (endolysosomes) and the cytosolic route (proteasomes) was profoundly impacted (Figure 1). Shown in Supplementary Table 1 is a list of gene descriptions and fold changes of all presented genes selected for the heatmap of Figure 1a. Interestingly, expression of MR1, a receptor involved in the activation of mucosal-associated invariant T cells (MAIT) by presentation of metabolites of vitamin B,^13^ was also strongly upregulated in B-LCLs (data not shown).

Collectively, this RNA sequencing data highlights how LCV infection induces a unique transcription profile that is markedly different from non-infected CD20^+^ B cells. This unique transcript profile, with enhanced expression of key co-stimulatory molecules and altered proteasome and endolysosome function, indicates that the LCV-infected B cell is an atypical APC. Of note, LCV infection also endows B cells with the ability to rescue proteolysis-sensitive self-antigens from destructive processing via citrullination as previously demonstrated,^14^ which may be involved in the association between autoimmune disease and progression of primate EAE.

### A dichotomous influence of B-LCL on T-cell homing receptor CCR7

**Figure 2:**
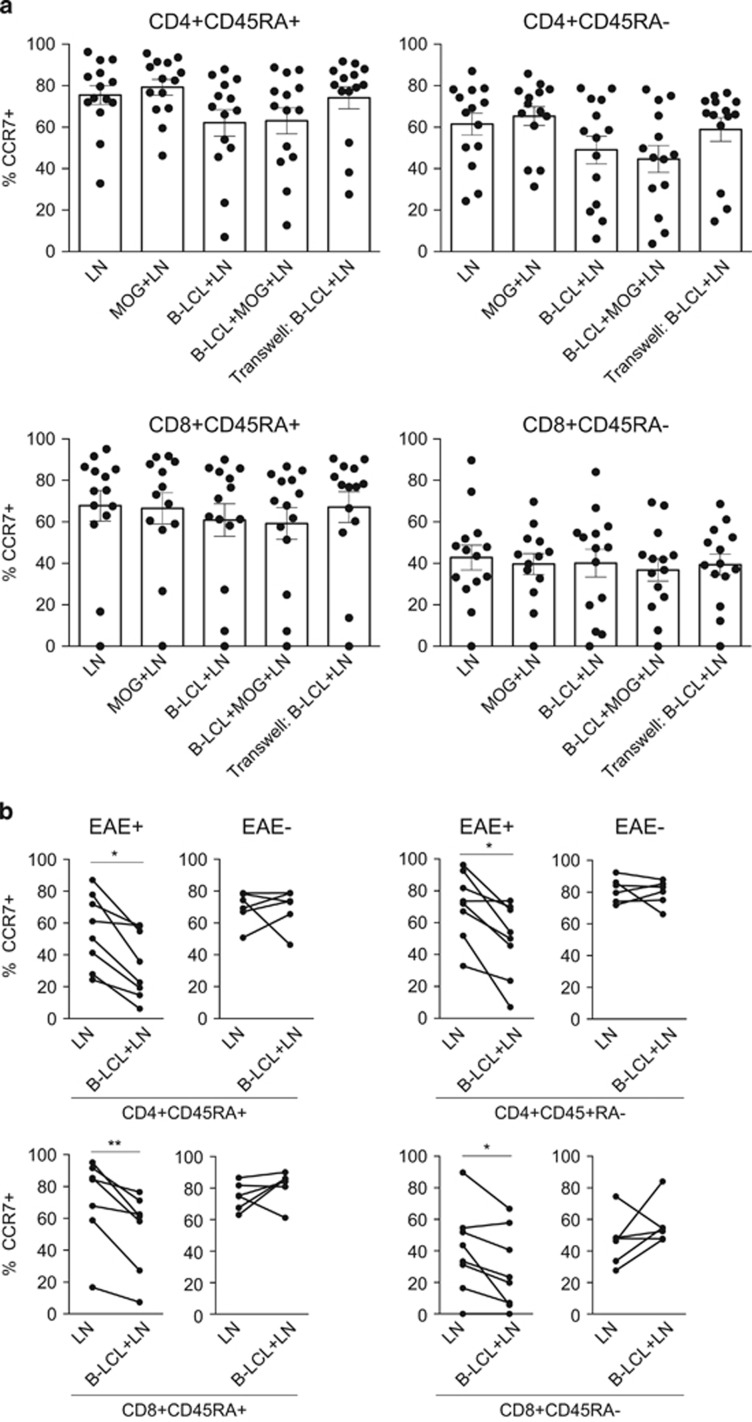
A dichotomous effect of B-LCLs on T-cell homing receptor CCR7. Mononuclear cells derived from the axillary lymph nodes (LNs) of marmosets (*n*=14) immunized with MOG34–56/IFA were co-cultured with CellTrace-labeled B-LCLs with or without the addition of MOG34–56 for 48 h. A transwell system in which B-LCLs and LNs were separated was used as a control (no MOG34–56 was added). Expression of T-cell homing receptor CCR7 was analyzed by flow cytometry following exclusion of CellTrace-labeled B-LCLs and dead cells. Lymphocytes were selected based upon forward scatter/side scatter selection and T-cell subsets were identified for the expression of CD3^+^, CD4^+^ or CD8^+^ and CD45RA^+/−^. Data are presented as % CCR7-expressing cells from the parent gate indicated above or below the graphs. (**a**) Co-culture with B-LCLs had no overall effect on the percentage of CD3^+^CD4^+^CD45^+/−^ and CD3^+^CD8^+^CD45^+/−^ cells expressing CCR7. (**b**) When data were analyzed based upon clinically evident EAE (EAE^+^) in comparison to monkeys that failed to develop EAE (EAE^−^), a reduced expression of the percentage of CD3^+^CD4^+^CD45^+/−^ and CD3^+^CD8^+^ CD45^+/−^ cells expressing CCR7 was observed in co-cultures of EAE^+^ animals. Reductions in CCR7^+^ T cells in EAE^+^ monkeys were not restricted to CD45RA^−^ (memory cells) or CD45RA^+^ (effector cells), and did not require MOG34–56 stimulation. Statistical significance (*P*<0.05) is indicated by an asterisk, significance of *P*<0.01 is indicated by **.

Previous reports implicating the CalHV-3^+^/EBV-infected B cell as the license for T-cell egression from the lymph node warrant further investigation.^15^ Here we assessed the effect of B-LCLs on the expression of CCR7, which was previously known as EBV-induced molecule 1 and is a vital receptor in controlling the dynamic lymphocyte homing towards secondary lymphoid organs (SLOs).^16, 17, 18^ Overall, B-LCLs had no consistent effect on CCR7 expression on CD4^+^ or CD8^+^ T-cell subsets in either CD45RA^−^ memory cells or CD45RA^+^ naive cells (Figure 2a). As we noticed reduced expression of CCR7 in some animals, we analyzed the data based on whether the mononuclear cell (MNC) donor animals had developed clinically evident EAE. Indeed, co-culture with B-LCLs induced significant reduction of CCR7^+^ T cells in MNC isolated from animals that had developed clinically evident EAE (score ⩾2.5), whereas no effect was observed on the percentage of CCR7^+^ T cells in MNC isolated from marmosets that failed to develop clinically evident EAE (Figure 2b).

Reductions in CCR7 were observed in the presence or absence of MOG34–56, indicating that the effect is independent of antigen recognition (Figure 2a). Moreover, a similar effect was observed in CD4^+^ and CD8^+^ T cells irrespective of the expression of CD45RA, indicating that this effect is not restricted to effector, memory or naive populations (Figure 2b).

### B-LCLs induce profound reduction of CD27 expression and alter Th17 responses

**Figure 3:**
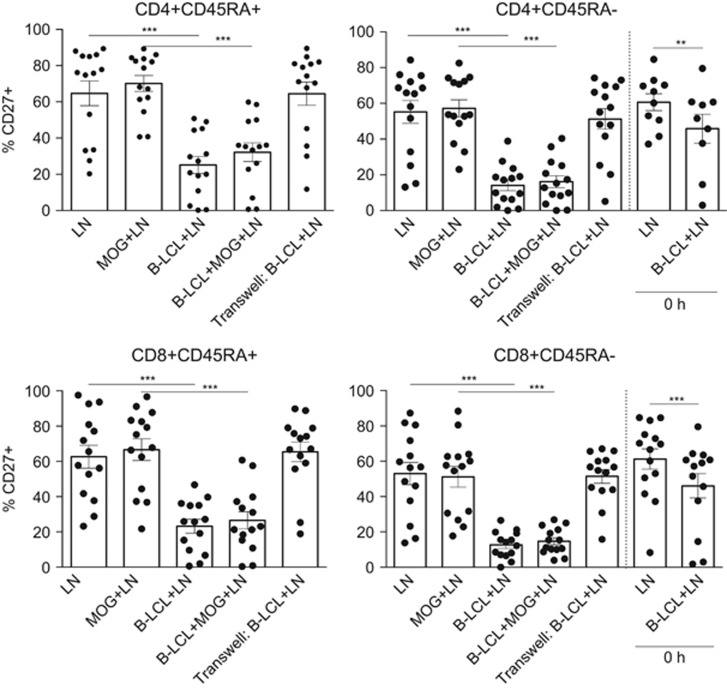
Tumor necrosis factor family member CD27 is lost after co-culture. Mononuclear cells derived from the axillary lymph nodes (LNs) of marmosets (*n*=14) immunized with MOG34–56/IFA were co-cultured with CellTrace-labeled B-LCLs with or without the addition of MOG34–56 for 48 h. A transwell system in which B-LCLs and LNs were separated was used as a control (no MOG34–56 was added). Shown is the percentage of CD27-expressing cells within the parent gate indicated above the graphs. The percentage of CD27-expressing cells was severely reduced upon co-culture with B-LCLs (48 h). This was irrespective of naive or effector status or addition of MOG34–56 and already occurred at 0 h measurement for memory (CD45RA^−^) cells as denoted by 0 h bar. Statistical significance of *P*<0.05 is indicated by an asterisk, significance of *P*<0.01 is indicated by **, significance of *P*<0.001 is indicated by ***.

EBV infection induced profound changes in the expression of co-stimulatory molecules by B cells. One peculiar, and often overlooked, feature of EBV-infected B cells is an enriched expression of CD70.^19^ Similar to human B-LCLs, marmoset B-LCLs exhibit elevated expression of CD70 compared to non-infected CD20^+^ primary B cells (Figure 1). We investigated whether increased expression of CD70 would alter the expression of its counter-structure CD27 on marmoset T cells as was previously reported for human cells.^20^ Indeed, interaction with EBV-infected B-LCLs had a profound impact on the expression of CD27, both on CD4^+^ and CD8^+^ T cells (Figure 3). This effect was irrespective of MOG34–56 and not constrained to CD45RA expression, suggesting that this effect is not specific for only memory or naive T cells. In addition, cell–cell contact was essential as in the transwell cultures no reduction of CD27 was observed (Figure 3). As a control, we also analyzed cellular phenotypes at time point 0 h, where B-LCLs and MNCs were immediately processed for staining upon co-culture. Reductions in CD27 expression were observed within minutes after B-LCLs and MNCs were encountered, indicating that this phenomenon can also occur without extensive rounds of cell division or chronic stimulation (Figure 3).

**Figure 4:**
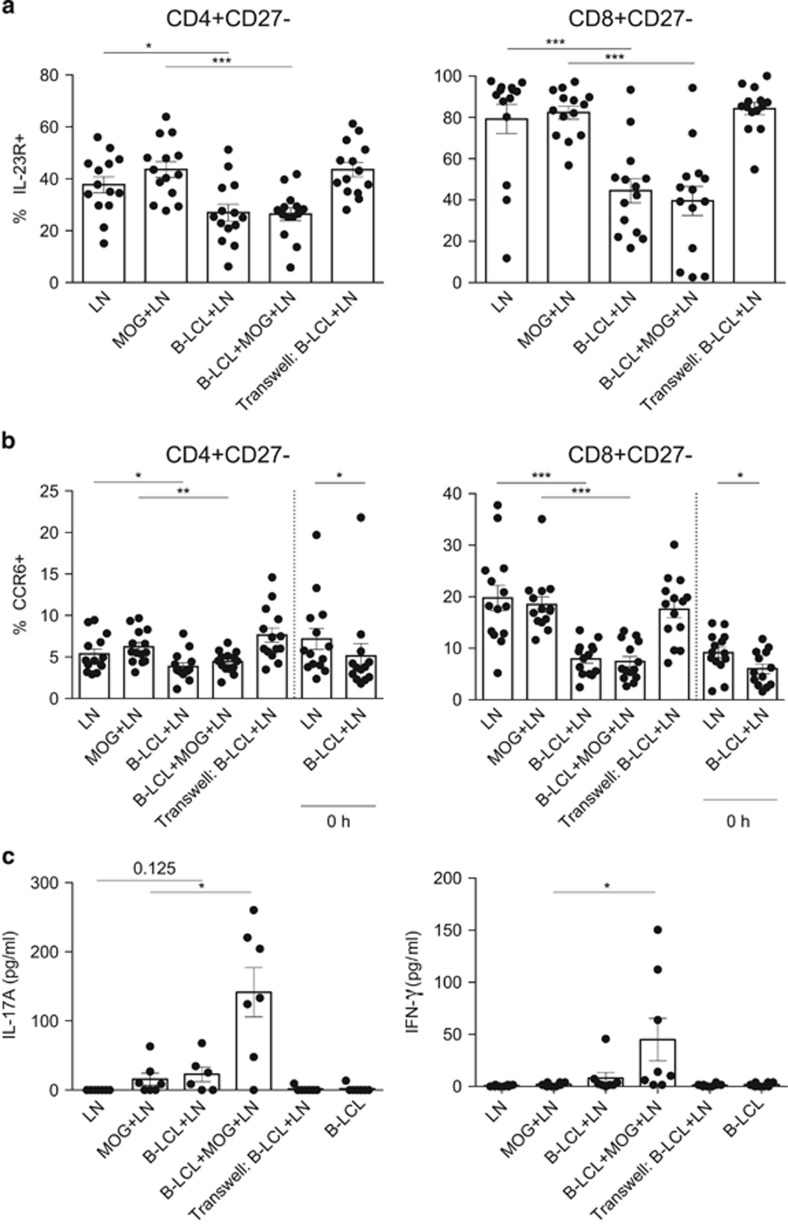
B-LCLs modulate Th17 responses. The effect of B-LCLs on Th17 responses was analyzed by classic Th17-associated markers by FACS and with the ProcartaPlex Luminex assay. Mononuclear cells derived from the axillary lymph nodes (LNs) of marmosets (*n*=14) immunized with MOG34–56/IFA were co-cultured with CellTrace-labeled B-LCLs with or without the addition of MOG34–56 for 48 h. A transwell system in which B-LCLs and LNs were separated was used as a control (no MOG34–56 was added). Supernatant was collected to measure cytokine production and cells were analyzed by FACS. Shown is the percentage of cells expressing either IL-23R (**a**) or CCR6 (**b**) from the parent gate indicated above the graphs. (**a** and **b**) Reductions in the percentage of IL-23R- and CCR6-expressing CD4^+^ and CD8^+^ T cells were observed in cells that had lost CD27 expression. In both cases, the addition of antigen was not required for reductions, and not limited to effector populations (data not shown). (**b**) Reductions in CCR6 were observed at initial co-culture periods (0 h) taken for baseline measurements. (**c**) Stimulation of B-LCL and antigen elicited significantly higher levels of IL-17A and IFN-γ compared to their respective controls following 48 h of co-culture. Secretions of both cytokines were dependent upon the addition of antigenic peptide. Detection of IL-23p19, IL-12p70 and IL-2 was absent after 24 and 48 h (data not shown). Statistical significance of *P*<0.05 is indicated by *, significance of *P*<0.01 is indicated by ** and *P*<0.001 is indicated by ***.

The intrinsic importance of the tumor necrosis factor receptor family member CD27 in the induction of Th17 responses has recently been detailed.^21^ We therefore assessed CCR6, IL-23R and Th1/Th17 cytokine expression following exposure to the B-LCLs (Figure 4). Significantly reduced expression levels of both CCR6 and IL-23R were found on CD4^+^ and CD8^+^ subsets of CD27^−^ cells (Figures 4a and b). This effect was observed regardless of CD45RA expression (data not shown), and was not dependent on the presence of the MOG34–56 peptide. Although we did not observe impacts on IL-23R expression at early time points (data not shown), significant reductions of CCR6 expression on CD27^−^ T cells were observed shortly after cells were brought together (Figure 4b).

Supernatants of co-cultures of MNC with B-LCL in the presence or absence of MOG34–56 were analyzed for secreted cytokines. Negligible production of IL-23p19, IL-12p70 and IL-2 was observed after 24 and 48 h incubation (data not shown). Interestingly, although reductions of CD27, CCR6 and IL-23R in co-cultures were observed regardless of addition of MOG34–56 (Figures 4a and b), the addition of B-LCLs and MOG34–56 to MNCs elicited significantly higher levels of IL-17A than in MNC cultures with only B-LCL or MOG34–56 (Figure 4c). The addition of B-LCLs with MOG34–56 also elicited significant increase of IFN-γ production compared to MOG34–56 stimulated MNCs without B-LCLs (Figure 4c). These effects were mitigated with transwell separation showing that direct cell–cell contact and antigen recognition are both required for cytokine production despite observations of changes at the surface marker level.

### Modulation of CD127 and CD95 is mediated by cell–cell contact and not by IL-7 secretion

**Figure 5:**
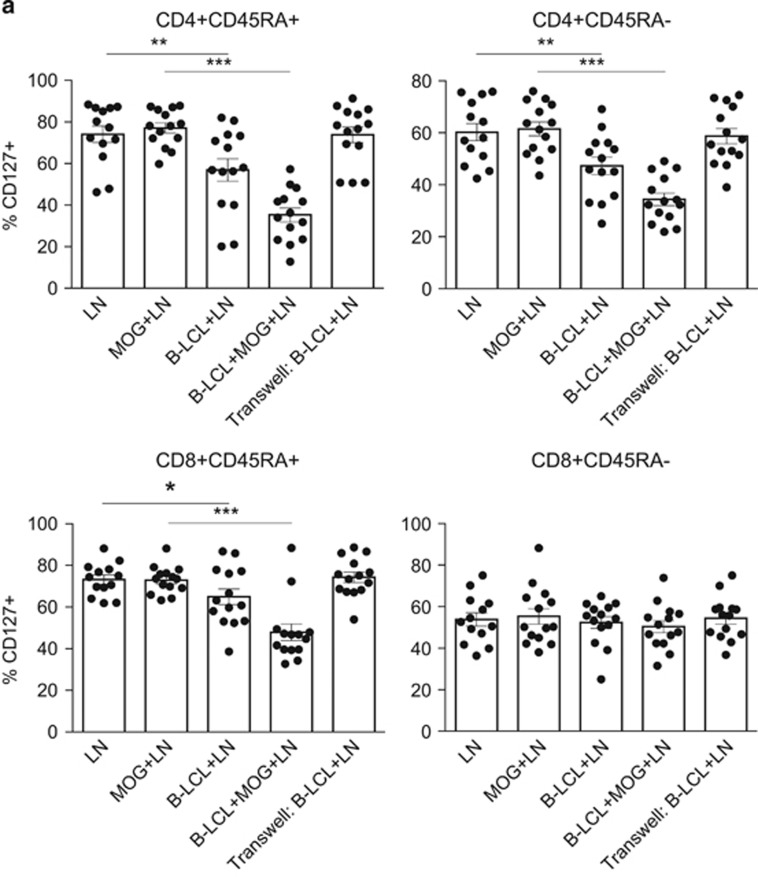

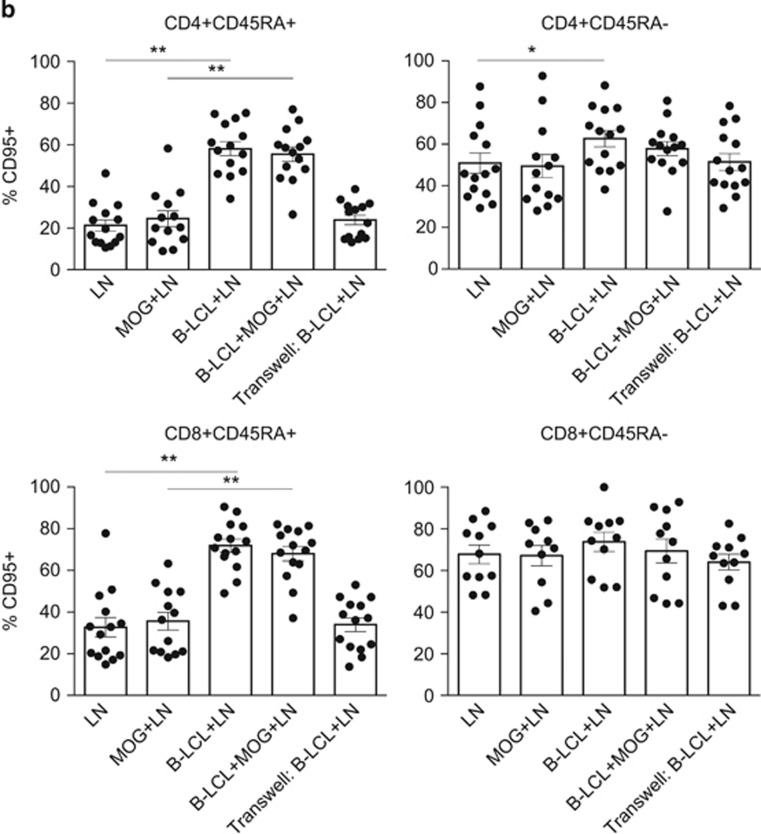
Modulation of CD127 and CD95 is mediated by cognate B–T interaction and not by IL-7 secretion. (**a**) Co-culture of mononuclear cells (MNCs) from axillary lymph node (LN; *n*=14) with B-LCLs resulted in minor reductions in the percentage of CD3^+^CD4^+^/CD8^+^CD45RA^+^ cells expressing CD127 (IL-7R). Reductions were greater with the addition of antigen (MOG34–56). This effect was also observed in memory subset (CD45RA^−^) in CD4^+^ T cells, but not CD8^+^ T cells. Physical separation of cell populations by porous membrane, that would enable cytokine diffusion, mitigated reductions of CD127. (**b**) B-LCLs induced a significant increase in the expression of CD95 in effector and naive (CD45RA^+^) T-cell populations, but less so in memory subsets. In naive and effector populations, this effect was not restricted to CD4/CD8 expression, did not require antigen stimulation and was also mitigated by transwell system. Statistical significance of *P*<0.05 is indicated by *, significance of *P*<0.01 is indicated by ** and *P*<0.001 is indicated by ***.

It has been demonstrated that EBV-transformed human B-cell lines constitutively secrete the cytokine IL-7 and express CD95L (FasL).^22, 23^ Our own data also indicate that marmoset B-LCLs express CD95L (Figure 1a), and that the IL-7/CD127 pathway has been implicated in marmoset EAE progression.^24^ A relationship between IL-7 and CD95 (Fas) expression has been established^25, 26^ and because of these data, we hypothesized that co-culture with the B-LCLs affects both IL-7R (CD127) and CD95 expression on T cells. We observed profound reductions of CD127 both in CD4^+^ and CD8^+^ populations. The reduction of CD127 occurred regardless of CD45RA expression in CD4^+^ T cells, but was not observed in CD45RA-expressing cells in the CD8^+^ compartment (Figure 5a). The addition of antigen (MOG34–56) resulted in more profound reductions in CD4-expressing T cells, and was a requirement for CD127 reductions in CD8-expressing T cells (Figure 5a). Reductions of CD127 depended on physical contact between B-LCLs and T cells, indicating that reduced detection of CD127 was not cytokine mediated or due to receptor occupancy by constitutively secreted IL-7.

The addition of the B-LCLs led to significantly increased CD95 expression in CD45RA-expressing CD4^+^ and CD8^+^ T cells, but only had a negligible impact on CD95 expression by CD45RA^−^ T cells (Figure 5b). Unlike CD127, altered expression of CD95 was not strongly influenced by addition of antigen to the culture. No alterations in the expression of CD127 or CD95 were observed when the B-LCLs and T cells were physically separated by porous polyethylene terephalate membranes, which would allow diffusion of cytokines from the top to bottom chambers (Figures 5a and b).

### B-LCL stimulates profound expression of CD279 (PD-1)

The data discussed thus far demonstrate that T-cell exposure to B-LCLs alters the expression of a variety of factors, many of which are not restricted by the addition of MOG34–56 antigen and which are induced by the contact with B-LCLs. Cells chronically infected with herpes virus (cytomegalovirus (CMV) and EBV) and malignant cells employ mechanisms of immune evasion, such as alterations in the CD279/CD274 signaling pathway and the interaction of CD70 with CD27.^27, 28^ In mouse melanoma models, anti-CD27 treatment correlated to lower CD279 expression on CD8^+^ T cells. On the other hand, overexpression of CD70 in dendritic cells is sufficient to break CD279 (PD-1)-mediated tolerance in ovalbumin-immunized CD11c-*CD70*tg mice.^29, 30, 31^

**Figure 6:**
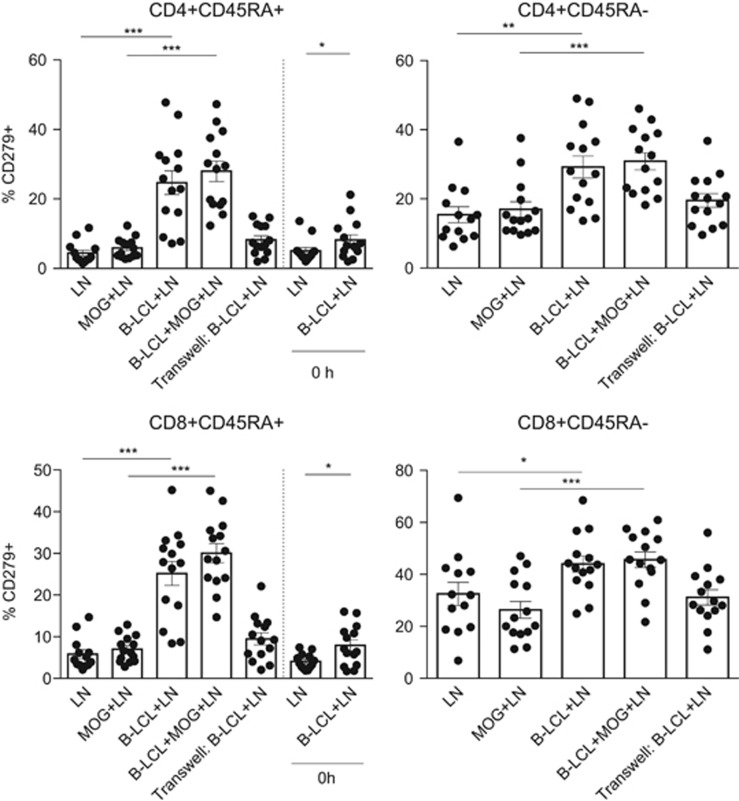
B-LCL rapidly alters CD279 (PD-1) expression in T-cell subsets. Expression of CD279 (PD-1) on MNC-derived T cells (*n*=14) was analyzed by flow cytometry following co-culture with B-LCLs. The figure depicts percentages of CD3^+^CD4^+^CD45RA^+/−^ (top panel)- and CD3^+^CD8^+^CD45RA^+/−^ (bottom panel)-expressing CD279. In all T-cell subsets examined following 48 h of co-culture, profound increases in the percentage of CD279^+^ cells were observed. In earliest time points measured (0 h) taken for calibration purposes, whereby cells were co-cultured and immediately centrifuged and stained for FACS, expression of CD279 was also increased in either CD4 or CD8 T-cell subsets expressing CD45RA. Statistical significance of *P*<0.05 is indicated by *, significance of *P*<0.01 is indicated by ** and *P*<0.001 is indicated by ***.

We assessed the expression of CD279 on T cells upon co-culture with B-LCLs. Shown in Figure 6 is the significant increase in the percentage of T cells expressing CD279. Upregulation of PD-1 expression was observed regardless of CD45RA expression in both CD4 and CD8-expressing T cells on this parameter. Moreover, addition of MOG peptide in co-culture conditions only had a minor effect. Despite previous associations between CD27/CD70 and PD-1 expression, CD279 increase was not restricted to CD27^−^ T cells and thus no relationship between CD279 and CD27 expression was observed in this system (data not shown).

### T cells acquire APC-associated co-stimulatory markers

**Figure 7:**
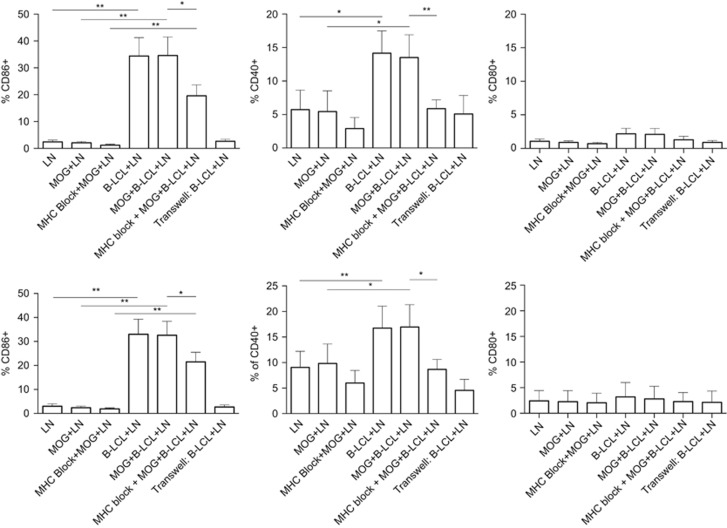
B-LCL-mediated T-cell acquisition of APC markers. Short-term co-cultures of mononuclear cells derived from the axillary lymph nodes (LNs) of marmosets (*n*=14) immunized with MOG34–56/IFA with B-LCLs (3 h) resulted in detection of CD86 and CD40 expression, but not CD80, on both CD4 (top panel) and CD8 (bottom panel) T-cell populations. This effect required cognate interaction between B-LCLs and T cell as the addition of antibodies to block of MHC class I and MHC class II significantly reduced detection of both CD86 and CD40. Statistical significance of *P*<0.05 is indicated by *, significance of *P*<0.01 is indicated by ** and *P*<0.001 is indicated by ***.

During the cognate interaction of T cells with malignant cells, membrane patches can be exchanged, a phenomenon known as trogocytosis. As an example, trogocytosis drives the acquisition of CD86 and HLA-G by T cells as an immune evasive mechanism in multiple myeloma patients.^32^ We examined whether evidence for trogocytosis could be found in the interaction of T cells with B-LCLs from marmosets and whether this may explain the acquisition by T cells of CD86 observed in longer co-culture experiments (own unpublished data). Shown in Figure 7 is the expression of co-stimulation molecules of APCs (CD80, CD86 and CD40) on CD4^+^ and CD8^+^ T cells following a 3 h co-culture with B-LCLs. Interestingly, we observed only transfer of CD40 and CD86, not of CD80. Moreover, this effect was, in part, mitigated by the addition of MHC I and II blocking antibodies and completely mitigated by physical separation in a transwell system. Acquisition of co-stimulatory signals via trogocytosis was observed in both CD4^+^ and CD8^+^ T-cell subsets (Figure 7). The absence of detectable CD80 transfer may be an artifact of time point measured.^33^ Unfortunately, technical limitations with fluorescence-activated cell sorting (FACS) sorting marmoset cells of high viability and the very limited cell number available for assays precluded further exploration of the functionality of this phenomenon.

## Discussion

A mechanistic explanation for the well-documented serological and epidemiological link between EBV and MS^34, 35, 36^ eludes scientists studying the immunobiology of MS risk factors. Research in a translationally relevant non-human primate model for MS, EAE in common marmosets, demonstrated a central pathogenic role of LCV-infected B-LCL (review^37^). This was concluded, among others, from the distinct clinical effect of therapeutic anti-CD20 mAbs versus mAbs against the B-cell growth and differentiation factors BLyS/BAFF and APRIL, which could be explained by differential depletion of the LCV-infected B-cell subset.^38^ Follow-up studies demonstrated that B-LCLs have a crucial role in the immune mechanisms driving EAE progression, namely, the activation and licensing for SLO egression of the autoreactive T cells and cross-presentation of self-antigen to auto-aggressive cytotoxic T lymphocyte (CTL).^15^ The current study was undertaken to gain further insight into messages exchanged in the *ex vivo* cross-talk between B-LCLs and (autoreactive) T cells.

We first examined the effect of EBV infection on the expression of B-cell genes relevant to T-cell activation and potentially involved in the cognate interaction with the T cell. In addition, we performed *ex vivo* co-culture assays with MNCs isolated from the SLO of MOG34–56/IFA-immunized marmosets and EBV-infected B-LCL generated prior to induction of EAE. Assays were performed in the presence and absence of the immunizing MOG34–56 peptide to be able to distinguish general effects on T cells from those exchanged in the cognate interaction between B-LCLs and autoreactive T cells.

The concept that LCV-infected B cells act as professional APCs for the pathogenic T cells is in itself a paradox. It implies that the infected B cells simultaneously trigger a T-cell response against the immunizing peptide MOG34–56 as well as avoid detection by anti-viral T cells for escaping a cytotoxic response. RNA sequencing data presented here demonstrate that the expression of many key genes involved with communication to the T cell is altered upon infection with LCV. Another explanation may be the observation that the autoreactive T cells recognize the epitope MOG40–48 of the auto-aggressive CTL in the context of major histocompatibility complex (MHC) class I/Caja-E molecules.^39^ MHC-E molecules have a well-documented role in immune escape from herpes virus-infected T cells.^40^ This dual role of MHC-E depends on the type of the peptide bound in the cleft, which directs interaction either to stimulatory CD94/NKG-2C or inhibitory CD94/NKG-2A complexes.^41^

Evidence suggests that one of several contributions of the B-LCL in EAE progression is the activation and licensing for SLO egression of the (autoreactive) T cells.^15^ Binding of chemokines such as CCL19 and CCL21 to CCR7 directs T-cell homing to SLO and reduction in CCR7 expression gives T cells the license to egress from SLO, after which they can migrate to their target organ. These data, in scope of previous reports using B-cell depletion with clinically relevant mAbs (ofatumumab and belimumab), support a role of the LCV-transformed B cell as the license for T-cell egression from the SLO.^9^ Intriguingly, we show here downregulation of the SLO homing receptor CCR7 by B-LCL only in T cells from clinical EAE responder marmosets (EAE score ⩾2.5) indicating that the downregulation of CCR7 may be a rate-limiting step in the pathogenic process. Another explanation for this phenomenon would be that T cells from marmosets with clinically evident EAE have enhanced activity or an altered activation state. However, this dichotomous response to B-LCLs was not observed for the other markers analyzed in the current study.

EBV-specific human T cells express CD27 and homozygous mutations in CD27 resulting in CD27 deficiency are associated with persistent EBV viremia, suggesting that the CD27/CD70 axis plays an important role in the control of EBV infection.^42, 43^ It is well possible that the high CD70 expression by the EBV B-LCLs, which is confirmed by our RNAseq data, supports immune escape, as seen in human glioblastomas and in lymphoproliferative diseases.^44^ Other evidence indicates that the CD27/CD70 pathway plays an important pro-pathogenic role in autoimmune conditions. Ligation of CD70 to CD27 induces release of soluble CD27 (sCD27) and it is indeed interesting that increased soluble CD27 level is a hallmark of diverse autoimmune conditions with a strong link to EBV, such as in cerebrospinal fluid in MS, in synovial tissue in RA and in serum in both systemic lupus erythematosus and Sjögren's syndrome.^22, 45, 46, 47, 48^ We propose that the elevated CD70 expression is an important, overlooked feature of EBV-infected B cells and that the CD27/CD70 axis may be relevant in the association between EBV and MS.

The observation that B-LCLs pulsed with MOG34–56 induce IL-17A is interesting, as the reduction of CD27, CCR6 and IL-23R occurred independent from addition of the immunizing peptide. This finding may indicate that physical interaction between B-LCLs and T cells is sufficient to alter surface markers expression on the T cell, whereas presentation of the antigenic peptide was required to elicit a functional response, that is, expression of IL-17A, which is the cytokine signature of the EAE model induced with MOG34–56/IFA.^11^

The here-reported data show that cross-talk with B-LCLs induces enhanced PD-1 expression on T cells. This is potentially relevant for MS, regarding the well-documented role of PD-1 and its ligand PD-L1 in the regulation of T-cell immunity and tolerance.^31^ It has been suggested and supported by evidence that autoimmune disease results from a general dysfunction of immune homeostatic mechanisms and peripheral tolerance breakdown.^49^ There is an established correlation between EBV viral load in acute infectious mononucleosis and PD-1 expression. Furthermore, the PD-1/PD-L1 pathway is altered in MS and elevated PD-1/PD-L1 expression has been associated with disease remission in MS patients.^50, 51^ It is tempting to speculate that repetitively enhanced expression of PD-1 (that is, receptor exhaustion), possibly as an immune escape mechanism for B-LCLs, would have consequences for subsequent PD-1 functionality or expression.

Our results confirm previous reports showing enhanced expression of CD95 in response to B-LCLs.^52^ In murine transgenic systems, CD27–CD70 interactions sensitize T cells for CD95-mediated apoptosis.^53^ Elevated expression of CD95L, a feature of marmoset B-LCL, has also been shown to upregulate CD95 expression. Although the B-LCLs constitutively secrete IL-7, and IL-7 has been shown to upregulate CD95 expression, altered expression of CD95 was dependent on cell–cell contact in our test system. Hence, the effect may be mediated through CD70 and/or CD95 stimulation. Given the biological importance of the CD95/CD95L pathway in the containment of T-cell responses, any alteration of this pathway has potential relevance to MS. The T-cell CD95/Fas function is dysregulated in the MS patient with increases in soluble CD95 in relapsing remitting MS and elevated CD95L expression in inflammatory infiltrates in both active and chronic lesions.^54, 55^ It may be that modulation of CD95 is just representative of activation, or possibly suppression through apoptosis, as previously suggested.^23^ The biological plausibility of this association is not a leap as one hallmark of acute infectious mononucleosis is enhanced CD95 and CD95L expression.^52, 56^

The current study also shows expression of co-stimulatory molecules of APC on T cells, CD40 and CD86, at much earlier time points than shown by others. This effect may be attributable to the transfer of membrane patches during cognate interaction of APCs and T cells, a phenomenon known as trogocytosis.^57, 58^ Although we did not observe CD80 transfer, this could be a kinetics issue as previously noted.^33^ It has been shown that EBV utilizes trogocytosis as a strategy to endow cells with receptors important for viral transmission. However, the functional significance of trogocytosis itself is debated and suggested effects seem wide-ranging; implication of potential regulatory effects on the T cell of the MS patient warrant further investigation.^59, 60^ The transfer of CD40 in particular is interesting given the importance of CD40 in functional CD8 T-cell responses (that is, cytokine secretion and proliferation), and evidence suggesting that these CD4–CD8 collaborations can be dissociated from APC help; acquisition of CD40 by CD8+ T cells could allow CD4 cells to directly help CD8 cells via CD40–CD40L interaction.^58^ Moreover, recently it has been observed that a subset of CD4+ T cells expressing CD40 are expanded in MS and serve as a new biomarker for disease.^61^

In conclusion, we propose that the LCV-infected B cell may play a role in autoimmune disease as an atypical APC, which is highlighted by enhanced expression of co-stimulatory markers such as CD70 and CD95L. Correlations between multiple autoimmune diseases, a lack of identification of EBV in disease-specific target tissues and temporal relationship between elevated levels of EBV in relation to disease activity provide strong arguments for this line of thinking. Although it could be argued that EBV infection is a by-product of abnormal dysfunctional immune responses due to disease activity, these results highlight the profound impact B-LCL (that is, constitutively CD70- and CD95L-expressing APCs), can have on the SLO-derived T cell. This study demonstrates that EBV infection of the B cell alters the normal communication process between the T and B cell, and highlights potential roles in T-cell egression and activation and Th17 responses. Future investigations of these pathways in MS-derived materials may provide valuable insights in any role EBV may have in autoimmune disease.

## Methods

### Cell source

Consistent with the 3R principles of animal experimentation, this research was performed with surplus cells from scheduled EAE studies in which the efficacy of new therapies was tested. Due to the limited number of cells available from some marmosets, not all assays could be performed for all individuals. Marmosets treated with pharmacological agents were included in this study only after individual analysis of data found no discrepancies between placebo and treated animals. MNCs isolated from axillary lymph nodes (ALNs) were obtained from 14 adult (female, age ranging between 2 and 5 years) common marmosets (*Callithrix jacchus*) enrolled in EAE studies. All marmosets were acquired from the purpose-bred colony at the BPRC (Rijswijk, Netherlands) and declared in good health by veterinary staff prior to enrollment into the study. Health checks included physical examination and tests for hematological, serological and microbiological abnormalities.

The used EAE model was induced with a synthetic peptide representing residues 34–56 of human MOG (MOG34–56; Cambridge Research Biochemicals Ltd, Cleveland, UK, sequence=GMEVGWYRPPFSRVVHLYRNGKD) emulsified with IFA (Difco Labs, Detroit, MI, USA) as previously described.^62^

### Ethics

The EAE experiments from which MNC were derived for this research were reviewed and approved of by the Institute's Animal Ethics Committee. Marmosets were housed and handled according to the Dutch Law on animal experimentation. The animal facilities of BPRC have been inspected and accredited by AAALAC in full.

### Generation of EBV-infected B-LCLs

The B95-8 EBV strain, henceforth indicated as EBV, was isolated from a human with infectious mononucleosis and has been maintained for decades in marmoset lymphocytes.^63^ The virus infects human and marmoset B cells, providing a well-established and frequently used experimental model of EBV infection.

Four weeks prior to the start of the EAE studies B-LCLs were generated by the infection of blood MNCs with human EBV B95-8.^62^ Briefly, peripheral blood mononuclear cells were cultured with supernatant from the EBV-producing B95-8 cell line for 1.5 h at 37 °C. Following incubation, cells were diluted at a 1:1 ratio with RPMI-1640 (Gibco Life Technologies, Bleiswijk Netherlands) culture medium containing 1 μg ml^−1^ phytohaemagglutinin and supplemented with 10% v/v heat-inactivated fetal calf serum (Gibco), 2.0 mm l-alanyl-l-glutamine (Gibco) and 50.0 U ml^−1^ penicillin/streptomycin (Gibco). Following successful transformation, cells were maintained in culture medium without phytohaemagglutinin until further use. Cells were mycoplasma negative and exhibited no irregularities.

### Co-culture of MNCs with EBV-infected B cells

At necropsy of MNC donors, the (semi-) autologous B-LCLs were collected from culture, irradiated at 50 Gy to prevent proliferation and stained with 1.0 μm CellTrace Violet (Molecular Probes, Eugene, OR, USA) for flow cytometric identification. B-LCLs were mixed with MNCs (*n*=14) from the ALN (1:1) and incubated in RPMI-1640 (Gibco) culture media supplemented with 10% v/v heat-inactivated fetal calf serum, l-alanyl-l-glutamine (Gibco) and penicillin/streptomycin, and cells were plated directly in 96-well V-bottom plates for initial (0 h) flow cytometric analysis or in 24-well plates for longer incubation periods (48 h). Blocking of marmoset MHC molecules was performed with mAbs directed against human MHC class II (clone PDV5.2) and MHC class I (clone W6/3) at 1:100 ascites dilution after which cells were pulsed with MOG34–56 peptide.

Polyethlene terephalate cell culture inserts (1 μm pore; BD Falcon, Bedford, MA, USA) were used for transwell culture conditions, whereby B-LCLs and MOG34–56 were loaded into the top chamber, and MNCs were loaded into the bottom chamber (1:1). Flow cytometric-based assays for detecting trogocytosis were performed with 3 h co-culture periods and utilized MHC blocking and transwell systems as described above.

### Cellular phenotyping

MNCs were phenotyped by flow cytometry as previously described.^62^ Briefly, cells were stained with an LIVE/DEAD Fixable Aqua Dead Cell Stain (Invitrogen, Molecular Probes, Carlsbad, CA, USA) for 25 min at room temperature to exclude dead cells. An FcR blocking reagent was used to block nonspecific antibody staining (Miltenyi Biotec, Bergisch Gladbach, Germany). Subsequently, cells were stained with commercially available mAbs against human CD3 (SP34-2), CD4 (L200), CD27 (MT721), CD45RA (5H9), CD56 (NCAM 16.2), CD80 (L307.4), CD86 (IT2.2) and HLA-DR (L243; all from BD Biosciences, San Diego, CA, USA); CD279 (J105) and CD127 (ebioDR5) (both from Ebioscience, San Diego, CA, USA); IL-23R (218213), CCR6 (53103) and CCR7 (150503) (all from R&D systems, Minneapolis, MN, USA); CD20 (H299, Beckman Coulter, Pasedena, CA, USA); and CD8 (LT-8, Serotec, Dusseldorf, Germany). Cells were fixed in 1% cytofix (BD Biosciences) prior to measurement.

Flow cytometric measurements were performed utilizing the FACS LSRII fitted with FACSDiva 5.0 (BD Biosciences) and data were analyzed with FlowJo software (Treestar, Ashland, OR, USA). The gating strategy to determine phenotype and exclude B-LCLs in analysis was as follows: first, B-LCL was excluded by gating CellTrace-negative cells (=MNC). Next, lymphocytes were identified based on forward scatter and side scatter. Live cells were then selected as aqua viability stain negative cells. Within the living lymphocyte population T cells were selected based on the expression of CD3. Within the CD3^+^ T-cell population, CD4^+^CD8^−^ and CD4^−^CD8^+^ cells were distinguished. Within these subpopulations, additional markers, such as CD27 and CD45RA, were analyzed. Data in figures are presented as a percentage of the previous identifier gate. B-LCL controls were stained with CD3 and CD4/CD8, and gated as mentioned above, to ensure purity of analyzed T cells.

### Analysis of cytokine production

Cytokine production was analyzed with the ProcartaPlex Luminex assay (Ebioscience) containing the non-human primates IL-2, IL-23p19, IL-12p70 and IFN-γ, and the human IL-17A beads (bead numbers 19, 63, 34, 43 and 36, respectively). Briefly, 0.5 million MNCs (*N*=8 marmosets) were plated with equal number of B-LCL (1:1) in 0.5 ml of media as described above. Following 24 and 48 h of incubation at 37 °C, supernatants were isolated and stored at −20 °C. The Luminex multiplex assay was performed according to manufacturer's protocol and results were analyzed with the BioPlex 200 system (Bio-Rad, Hercules, CA, USA). Data were quantified based on manufacturer standards reconstituted in cell culture media.

### RNA sequencing

RNA sequencing was performed on spleen-derived CD20^+^ B cells isolated from healthy marmosets (*n*=2) and B-LCLs (*n*=2). Briefly, whole spleen homogenates from healthy control monkeys were stained with phycoerythrin labeled CD20 (B1-RD1; Beckman Coulter) and Horizon V500-labeled CD3 as described above. FACS-sorted CD3^−^CD20^+^ were stored as dry pellets at −80 °C.

RNA was isolated from CD3^−^CD20^+^ spleen cells and marmoset B-LCLs. Illumina HiSeq 2500 single-end RNA sequencing was performed at the Department of Genetics of the UMCG Groningen. For quality control, FASTQC was performed.^64^ Low-quality reads were trimmed with FASTX trimmer (Version 0.0.14).^65^ The hisat/0.1.5-beta-goolf-1.7.20 alignment^66^ was performed using *Callithrix jacchus* GRCm38.82 reference genome. Approximately 10 million high-quality uniquely mapped reads were obtained per sample. HT-seq count^67^ was utilized to quantify reads, which were imported into R (version 3.2.3) to be statistically analyzed for differential gene expression using EdgeR.^68^ Gene annotation and gene ontology data of the ensemble data were obtained using the R Biomart Bioconductor tool.^69^ A heatmap of log count per million values was generated using heatmap.2. Functional and pathway annotation of the differentially expressed genes was performed using Gene Ontology and Kegg from DAVID.^70, 71^

### Statistics

Statistical analysis was performed using Prism 6.0b for Mac OS X. Data were analyzed using the two-tailed paired Student's *t*-test (Wilcoxon). *P*-values<0.05 were considered statistically significant. Data are shown as individual values (*n*=14), although experiments were performed separately due to the variation in date of animal sacrifice. Error bars represent s.e.m.
