# Supplementary material for: Analysis of the cross-talk of Epstein–Barr virus-infected B cells with T cells in the marmoset
Source: Clin Transl Immunology. 2017 Feb 10;6(2):e127–. doi: 10.1038/cti.2017.1 (PMC5311918; doi:10.1038/cti.2017.1)
Supplement: Supplementary Table 1 [file cti20171x1.docx]

|  | **Gene** | **Gene Description** | **B-LCL vs CD20+ (Log 2 fold change)** |
| --- | --- | --- | --- |
| **Antigen Presentation** | CD70 | CD70 molecule | 7.79 |
|  | CD80 | CD80 molecule | 1.64 |
|  | CD86 | CD86 molecule | 2.11 |
|  | HFE | hemochromatosis | 2.47 |
|  | MR1 | major histocompatibility complex, class I-related | 3.38 |
|  | PDCD1 | programmed cell death 1 | 5.43 |
|  | RAB27A | roundabout, axon guidance receptor, homolog 3 (Drosophila) | 8.83 |
|  | FAS | Fas cell surface death receptor | 3.96 |
|  | FASLG | Fas ligand (TNF superfamily, member 6) | 2.51 |
|  | CD1B | CD1b molecule | -6.37 |
|  | CD1D | CD1d molecule | -5.59 |
|  | FCGRT | Fc Fragment Of IgG, Receptor, Transporter, Alpha | -4.41 |
|  | RAB32 | RAB32, member RAS oncogene family | -1.22 |
| **Lysosome** | ADA | adenosine deaminase | 1.37 |
|  | FASLG.1 | Fas ligand (TNF superfamily, member 6) | 3.96 |
|  | GAA | glucosidase, alpha; acid | 1.26 |
|  | GNS | glucosamine (N-acetyl)-6-sulfatase | 1.62 |
|  | LAMP2 | lysosomal-associated membrane protein 2 | 1.85 |
|  | LDLR | low density lipoprotein receptor | 4.43 |
|  | RAB9A | RAB9A, member RAS oncogene family | 1.11 |
|  | TMEM97 | transmembrane protein 97 | 1.85 |
|  | ACP5 | acid phosphatase 5, tartrate resistant | -6.44 |
|  | CTSH | cathepsin H | -2.72 |
|  | CTSK | cathepsin K | -5.52 |
|  | CTSS | cathepsin S | -1.54 |
|  | CTSW | cathepsin W | -4.68 |
|  | CYB561A3 | cytochrome b561 family member A 3 | -1.91 |
|  | HPSE | heparanase | -4.84 |
|  | LGMN | legumain | -2.89 |
|  | MARCH1 | membrane-associated ring finger (C3HC4) 1, E3 ubiquitin protein ligase | -2.63 |
|  | NAAA | N-acylethanolamine acid amidase | -2.64 |
|  | NEU1 | sialidase 1 (lysosomal sialidase) | -1.15 |
|  | NPC2 | Niemann-Pick disease, type C2 | -2.28 |
|  | RRAGD | Ras-related GTP binding D | -1.07 |
|  | SCARB2 | scavenger receptor class B, member 2 | -6.51 |
|  | UNC13D | unc-13 homolog D (C. elegans) | -5.11 |
| **Proteasome** | PSMD1 | proteasome (prosome, macropain) 26S subunit, non-ATPase, 1 | 1.15 |
|  | PSMD9 | proteasome (prosome, macropain) 26S subunit, non-ATPase, 9 | -1.07 |
|  | PSMC3 | proteasome (prosome, macropain) 26S subunit, ATPase, 3 | -1.08 |
|  | PSMG3 | proteasome (prosome, macropain) assembly chaperone 3 | -1.23 |
|  | SHFM1 | split hand/foot malformation (ectrodactyly) type 1 | -1.03 |

**Table S1.** List of all presented genes in Figure 1A represented as a fold change when comparing expression profiles of B-LCL vs CD20+.
